# Genome sequence of the model plant pathogen *Pectobacterium carotovorum* SCC1

**DOI:** 10.1186/s40793-017-0301-z

**Published:** 2017-12-20

**Authors:** Outi Niemi, Pia Laine, Patrik Koskinen, Miia Pasanen, Ville Pennanen, Heidi Harjunpää, Johanna Nykyri, Liisa Holm, Lars Paulin, Petri Auvinen, E. Tapio Palva, Minna Pirhonen

**Affiliations:** 1 Division of Genetics, Department of Biosciences, University of Helsinki, Helsinki, Finland; 2 Viikki Plant Science Centre, University of Helsinki, Helsinki, Finland; 3 Institute of Biotechnology, University of Helsinki, Helsinki, Finland; 4 Plant Pathology, Department of Agricultural Sciences, University of Helsinki, Helsinki, Finland

**Keywords:** *Pectobacterium*, Soft rot, Plant pathogen, Necrotroph, Potato, Finland

## Abstract

Bacteria of the genus *Pectobacterium* are economically important plant pathogens that cause soft rot disease on a wide variety of plant species. Here, we report the genome sequence of *Pectobacterium carotovorum* strain SCC1, a Finnish soft rot model strain isolated from a diseased potato tuber in the early 1980’s. The genome of strain SCC1 consists of one circular chromosome of 4,974,798 bp and one circular plasmid of 5524 bp. In total 4451 genes were predicted, of which 4349 are protein coding and 102 are RNA genes.

## Introduction

10.1601/nm.3241 species are economically important plant pathogens that cause soft rot and blackleg disease on a range of plant species across the world [1, 2]. The main virulence mechanism employed by 10.1601/nm.3241 is the secretion of vast amounts of plant cell wall-degrading enzymes [1, 3]. Due to their ability to effectively macerate plant tissue for acquisition of nutrients, 10.1601/nm.3241 species are considered classical examples of necrotrophic plant pathogens. Among the 10.1601/nm.3241 species, 10.1601/nm.10935 has the widest host range while potato is the most important crop affected in temperate regions [1, 4]. 10.1601/nm.10935 strain SCC1 was isolated from a diseased potato tuber in Finland in the early 1980’s [5]. It is highly virulent on model plant hosts such as tobacco (*Nicotiana tabacum*) and thale cress (*Arabidopsis thaliana*) as well as on the original host, potato (*Solanum tuberosum*). For three decades, the strain has been used as a model strain in the study of virulence mechanisms of 10.1601/nm.3241 as well as in the study of plant defense mechanisms against necrotrophic plant pathogens ([e.g. [6–13]). Here we describe the annotated genome sequence of 10.1601/nm.10935 strain SCC1.

## Organism information

### Classification and features

**Fig. 1:**
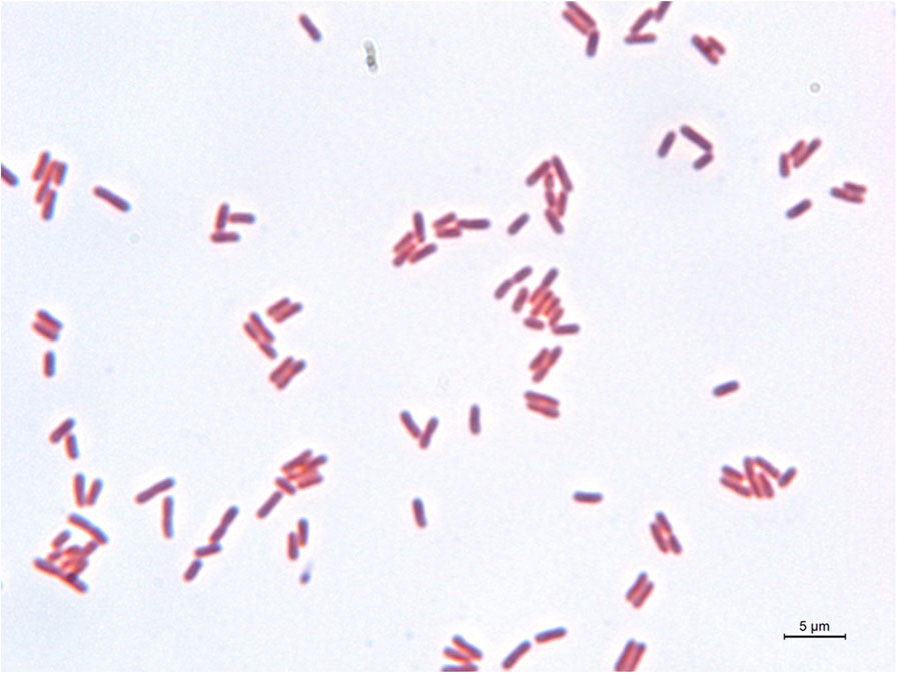
Photomicrograph of Gram stained exponentially growing *Pectobacterium carotovorum* SCC1 cells. A light microscope with 100× magnification was used

10.1601/nm.10935 strain SCC1 is a Gram-negative, motile, non-sporulating, and facultatively anaerobic bacterium that belongs to the order of 10.1601/nm.29303 within the class of 10.1601/nm.2068. Cells of strain SCC1 are rod shaped with length of approximately 2 μm in the exponential growth phase (Fig. 1). Strain SCC1 is pathogenic causing soft rot disease in plants. It was originally isolated from a diseased potato tuber in Finland in 1982 [5]. It also provokes maceration symptoms on model plants Arabidopsis, tobacco, and tomato (*Solanum lycopersicum*), and is used as a soft rot model in research.

**Fig. 2:**
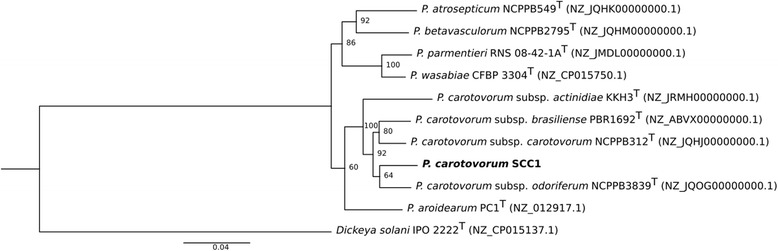
Maximum likelihood tree of *Pectobacterium carotovorum* SCC1 and other closely related *Pectobacterium* strains. The phylogenetic tree was constructed from the seven housekeeping genes (*dnaN, fusA, gyrB, recA, rplB, rpoS* and *gyrA*). The concatenated sequences were aligned using MAFFT multiple sequence alignment program (version 7) with default parameters [42]. The phylogenetic tree was built in RAxML (Randomized Axelerated Maximum Likelihood) program with Maximum likelihood (ML) inference [43]. 88 different nucleotide substitution models were tested with jModelTest 2.0 and the best model was selected using Akaike information criterion (AIC) [44]. Bootstrap values from 1000 replicates are shown in each branch. *Dickeya solani* IPO2222 was used as the outgroup. Type strains are marked with T after the strain name. GenBank accession numbers are presented in the parentheses. The scale bar indicates 0.04 substitutions per nucleotide position

Strain SCC1 has previously been described belonging to 10.1601/nm.3242 based on biochemical properties such as its ability to grow at +37 °C and in 5% NaCl, its sensitivity to erythromycin, its ability to assimilate lactose, melibiose and raffinose but not sorbitol, and its inability to produce reducing sugars from sucrose and acid from α-methyl glucoside [14]. A phylogenetic tree generated based on seven housekeeping genes (*dnaN, fusA, gyrB, recA, rplB, rpoS* and *gyrA*) clusters strain SCC1 together with other 10.1601/nm.10935 strains (Fig. 2). However, sequence based phylogenetic analysis was inconclusive regarding the subspecies status. Overall, the phylogeny of 10.1601/nm.3241 species and subspecies is currently in turmoil and assigning strains to subspecies is challenging [15].

10.1601/nm.10935 strain SCC1 has been deposited at the International Center for Microbial Resources - French collection of plant-associated bacteria (accession: 10.1601/strainfinder?urlappend=%3Fid%3DCFBP+8537). MIGS of strain SCC1 is summarized in Table 1.

**Table 1:** Classification and general features of *Pectobacterium carotovorum* strain SCC1 [46]

MIGS ID	Property	Term	Evidence code^a^
	Classification	Domain *Bacteria*	TAS [47]
		Phylum *Proteobacteria*	TAS [48]
		Class *Gammaproteobacteria*	TAS [49, 50]
		Order *Enterobacterales*	TAS [51]
		Family *Pectobacteriaceae*	TAS [51]
		Genus *Pectobacterium*	TAS [52, 53]
		Species *Pectobacterium carotovorum*	TAS [52, 54]
		Strain: SCC1 (10.1601/strainfinder?urlappend=%3Fid%3DCFBP+8537)	TAS [5]
	Gram stain	Negative	IDA
	Cell shape	Rod	IDA
	Motility	Motile	IDA
	Sporulation	Non-sporulating	NAS [51]
	Temperature range	Mesophile, able to grow at 37 °C	TAS [14]
	Optimum temperature	~28 °C	IDA
	pH range; Optimum	Unknown	
	Carbon source	Sucrose, lactose, melibiose, raffinose	IDA,TAS [14]
MIGS-6	Habitat	Potato	TAS [5]
MIGS-6.3	Salinity	Able to grow in 5% NaCl	TAS [14]
MIGS-22	Oxygen requirement	Facultatively anaerobic	NAS [51]
MIGS-15	Biotic relationship	Free-living	NAS
MIGS-14	Pathogenicity	Pathogenic	NAS [53]
MIGS-4	Geographic location	Finland	TAS [5]
MIGS-5	Sample collection	1982	NAS
MIGS-4.1	Latitude	60° 13′ 36.15” N	NAS
MIGS-4.2	Longitude	25° 00′ 54.77″ E	NAS
MIGS-4.4	Altitude	Unknown	

^a^Evidence codes - IDA: Inferred from Direct Assay; TAS: Traceable Author Statement (i.e., a direct report exists in the literature); NAS: Non-traceable Author Statement (i.e., not directly observed for the living, isolated sample, but based on a generally accepted property for the species, or anecdotal evidence). These evidence codes are from the Gene Ontology project [55]

## Genome sequencing information

### Genome project history

10.1601/nm.10935 strain SCC1 has been used as a model soft rot pathogen in the field of plant-pathogen interactions ever since its isolation in the 1980’s. The sequencing of the genome of strain SCC1 was initiated in 2008 in order to further facilitate its use as a model pathogen.

The project was carried out jointly by the Institute of Biotechnology, Department of Biosciences and Department of Agricultural Sciences at the University of Helsinki, Finland. The genome was sequenced, assembled and annotated. The final sequence contains two scaffolds representing one chromosome and one plasmid. The sequence of the chromosome contains one gap of estimated length of 3788 bp. The genome sequence is deposited in GenBank under the accession numbers CP021894 (chromosome) and CP021895 (plasmid). Summary information of the project is presented in Table 2.

**Table 2:** Project information

MIGS ID	Property	Term
MIGS 31	Finishing quality	One gap remaining, otherwise finished
MIGS-28	Libraries used	Standard 454 and Solid libraries
MIGS 29	Sequencing platforms	454, SOLiD, Sanger
MIGS 31.2	Fold coverage	Chromosome 40×, plasmid 67×
MIGS 30	Assemblers	gsAssembler v 1.1.03.24
MIGS 32	Gene calling method	Prodigal
	Locus Tag	SCC1
	Genbank ID	CP021894, CP021895
	GenBank Date of Release	July 27, 2017
	GOLD ID	
	BIOPROJECT	PRJNA379819
MIGS 13	Source Material Identifier	10.1601/strainfinder?urlappend=%3Fid%3DCFBP+8537
	Project relevance	Plant pathogen

### Growth conditions and genomic DNA preparation

After isolation from potato in 1982, 10.1601/nm.10935 strain SCC1 has been stored in 22% glycerol at −80 °C. For preparation of genomic DNA, the strain was first grown overnight on solid LB medium (10 g tryptone, 5 g yeast extract, 10 g NaCl, and 15 g agar per one liter of medium) at 28 °C. A single colony was then picked and grown overnight in 10 ml of liquid LB medium at 28 °C with shaking. Cells were harvested by centrifugation for 20 min at 3200 g at 4 °C and resuspended into TE buffer (10 mM Tris-HCl pH 7.5, 1 mM EDTA). SDS (5% *w*/*v*) and Proteinase K (1 mg/ml) were used to break the cells for one hour at 50 °C. Genomic DNA was extracted using phenol-chloroform purification followed by ethanol precipitation. The quantity and quality of the DNA was assessed by spectrophotometry and agarose gel electrophoresis.

### Genome sequencing and assembly

Genome sequencing was performed at DNA and Genomics Laboratory, Institute of Biotechnology, University of Helsinki, Finland. Genomic DNA was sequenced using 454 (454 Life Sciences/Roche), SOLiD3 (Life Technologies) and ABI 3130xl Genetic Analyzer (Life Technologies) instruments. DNA was fragmented into approximate size of 800 bp using Nebulizer (Roche) followed by standard fragment 454 library with the GS FLX series reagents. For the SOLiD library genomic DNA was fragmented with a Covaris S2 Sonicator (Covaris Inc.) to approximate size of 250 bp. The library was prepared using the SOLiD library kit (Life technologies).Newbler (version 1.1) was used to assemble 366,453 pyrosequencing reads (77,6 Mbp) in approximate length of 240 bp with default settings into 100 large (>1000 bp) contigs. GAP4 program (Staden package) was used for contig editing, primer design for PCRs and primer walking, and finishing the genome. Gaps were closed using PCR and traditional primer walking Sanger sequencing method. Finally, SOLiD reads were mapped to the genome and fifteen single genomic positions were fixed. Final sequencing coverages were 40× in genome and 67× in plasmid sequences.

### Genome annotation

Coding sequences were predicted using the Prodigal gene prediction tool [16]. GenePRIMP [17] was run to correct systematic errors made by Prodigal and to reanalyze the remaining intergenic regions for missed CDSs. Functional annotation for the predicted genes was performed using the PANNZER annotation tool [18]. The annotation was manually curated with information from publications and the following databases: COG [19], KEGG [20], CDD [21], UniProt and NCBI non-redundant protein sequences. To identify RNA genes, RNAmmer v1.2 [22] (rRNAs) and tRNAscan-SE [23] (tRNAs) were used. Clusters of Orthologous Groups assignments and Pfam domain predictions were done using the WebMGA server [24]. Transmembrane helices were predicted with TMHMM [25] and Phobius [26]. For signal peptide prediction, SignalP 4.1 [27] was used. CRISPRFinder [28] was used to detect Clustered Regularly Interspaced Short Palindromic Repeats (CRISPRs).

## Genome properties

**Fig. 3:**
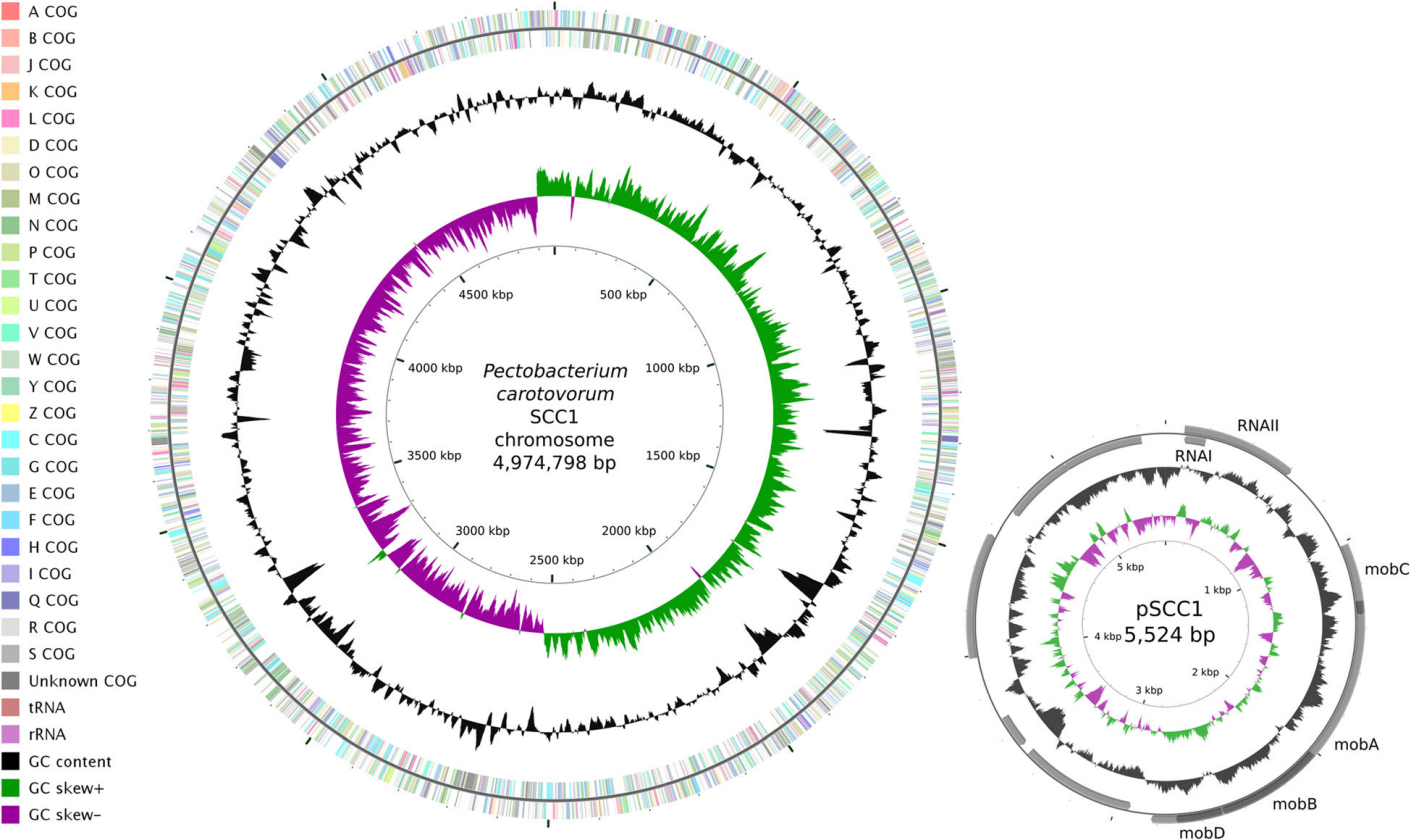
Circular maps of the chromosome and plasmid of *Pectobacterium carotovorum* SCC1. Rings from the outside to the center: Genes on forward strand (colored by COG categories), Genes on reverse strand (colored by COG categories), GC content, GC skew. Maps were generated using the CGView Server [45]

The genome of 10.1601/nm.10935 SCC1 consists of one circular 4,974,798 bp chromosome and one circular 5524 bp plasmid (Table 3, Fig. 3). The total genome size is 4,980,322 bp with an overall G + C content of 51.85% (Table 4). A total of 4451 genes were predicted, out of which 4440 are chromosomal and 11 reside on the plasmid. 4349 (97.71%) genes are protein coding and 102 (2.29%) are RNA genes (77 tRNA, 22 rRNA, and 3 other RNA genes). Of the 4349 protein coding genes, 3812 (87.65%) could be assigned to COG functional categories (Table 5).

**Table 3:** Summary of *P. carotovorum* SCC1 genome: one chromosome and one plasmid

Label	Size (Mb)	Topology	INSDC identifier	RefSeq ID
Chromosome	4.974798	Circular	CP021894	
Plasmid pSCC1	0.005524	Circular	CP021895

**Table 4:** Genome statistics

Attribute	Value	% of Total
Genome size (bp)	4,980,322	100.00
DNA coding (bp)	4,314,063	86.62
DNA G + C (bp)	2,580,564	51.85
DNA scaffolds	2	
Total genes	4451	100.00
Protein coding genes	4349	97.71
RNA genes	102	2.29
Pseudo genes	NA	NA
Genes in internal clusters	NA	NA
Genes with function prediction	3955	88.86
Genes assigned to COGs	3812	85.64
Genes with Pfam domains	3782	84.97
Genes with signal peptides	428	9.62
Genes with transmembrane helices	939	21.10
CRISPR repeats	5

**Table 5:** Number of genes associated with general COG functional categories

Code	Value	%age	Description
J	183	4.21	Translation, ribosomal structure and biogenesis
A	2	0.05	RNA processing and modification
K	332	7.63	Transcription
L	161	3.70	Replication, recombination and repair
B	0	0.00	Chromatin structure and dynamics
D	40	0.92	Cell cycle control, Cell division, chromosome partitioning
V	61	1.40	Defense mechanisms
T	242	5.56	Signal transduction mechanisms
M	241	5.54	Cell wall/membrane biogenesis
N	114	2.62	Cell motility
U	122	2.81	Intracellular trafficking and secretion
O	152	3.50	Posttranslational modification, protein turnover, chaperones
C	248	5.70	Energy production and conversion
G	376	8.65	Carbohydrate transport and metabolism
E	435	10.00	Amino acid transport and metabolism
F	94	2.16	Nucleotide transport and metabolism
H	177	4.07	Coenzyme transport and metabolism
I	103	2.37	Lipid transport and metabolism
P	318	7.31	Inorganic ion transport and metabolism
Q	67	1.54	Secondary metabolites biosynthesis, transport and catabolism
R	445	10.23	General function prediction only
S	358	8.23	Function unknown
–	537	12.35	Not in COGs

The total is based on the total number of protein coding genes in the genome

## Insights from the genome sequence

10.1601/nm.10935 strain SCC1 harbors a small cryptic plasmid of 5524 bp, pSCC1. The plasmid contains sequences for RNAI and RNAII, two non-coding RNAs involved in replication initiation and control in enterobacterial RNA priming plasmids such as ColE1 [29]. A similar replication region has previously been described in the small cryptic plasmid pEC3 of 10.1601/nm.3242 strain 10.1601/strainfinder?urlappend=%3Fid%3DIFO+3380 [30]. In addition to the two RNA genes, pSCC1 was predicted to contain nine protein-coding genes. Four of these (*mobABCD*) encode mobilization proteins. The *mob* locus is required for mobilization of non-self-transmissible plasmids and is found on many enterobacterial plasmids including pEC3 [31]. No function could be assigned to the remaining five genes on pSCC1. One of them, SCC1_4463, is very similar to genes found in many 10.1601/nm.3091 genomes, especially those of genera 10.1601/nm.3148, 10.1601/nm.3092 and 10.1601/nm.3291, whereas similar genes to the other four on pSCC1 are not widely present in other sequenced genomes.

10.1601/nm.3241 infection is characterized by maceration symptoms caused by the secretion of a large arsenal of plant cell wall-degrading enzymes. Accordingly, the genome of 10.1601/nm.10935 strain SCC1 was found to contain genes for eleven pectate lyases (*pelABCILWXZ*, *hrpW*, SCC1_1311, and SCC1_2381), one pectin lyase (*pnl*), four polygalacturonases (*pehAKNX*), one oligogalacturonate lyase (*ogl*), three cellulases (*celSV*, *bcsZ*), one rhamnogalacturonate lyase (*rhiE*), two pectin methylesterases (*pemAB*), and two pectin acetylesterases (*paeXY*). In addition, the genome harbors two genes encoding proteases previously characterized as plant cell wall-degrading enzymes (*prt1*, *prtW*) as well as a number of putative proteases, some of which may function in plant cell wall degradation. Different 10.1601/nm.3241 species and strains have been found to harbor very similar collections of plant cell wall-degrading enzymes [32], and the number and types of enzymes in the genome of strain SCC1 fit this picture well.

Protein secretion plays an essential role in soft rot pathogenesis [33]. The most important secretion system in 10.1601/nm.3241 is the type II secretion system, also known as the Out system (*outCDEFGHIJKLMN*), which transports proteins from the periplasmic space into the extracellular environment [34]. It is responsible for the secretion of most plant cell wall-degrading enzymes such as pectinases and cellulases as well as some other virulence factors such as the necrosis-inducing protein Nip [33, 35]. Furthermore, 10.1601/nm.3241 genomes typically harbor multiple type I secretion systems, which secrete proteases and adhesins [33]. At least four type I secretion systems are encoded in the genome of 10.1601/nm.10935 SCC1 (*prtDEF*, SCC1_1144–1146, SCC1_1589–1591, and SCC1_3286–3288). Strain SCC1 also harbors a type III secretion system cluster (SCC1_2406–2432), which has previously been characterized in this strain and shown to affect the speed of symptom development during infection [6, 36]. Overall, the role of the type III secretion system in 10.1601/nm.3241 is not well understood and 10.1601/nm.3254 and 10.1601/nm.29447 seem to lack it completely [32, 37]. The type IV secretion system has been shown to have a minor contribution to virulence of 10.1601/nm.3247 [38]. However, it is sporadically distributed among 10.1601/nm.3241 strains [33], and no type IV secretion genes could be found from the genome of 10.1601/nm.10935 SCC1. Finally, the type VI secretion system has also been shown to have a small effect on virulence at least in some 10.1601/nm.3241 species [32, 39]. In 10.1601/nm.10935 SCC1, one type VI secretion system cluster is present in the genome (SCC1_0988–1002).

Soft rot pathogens have been suggested to be able to use insect vectors in transmission, and indeed, certain 10.1601/nm.10935 strains can infect *Drosophila* flies and persist in their guts [40]. This ability has been linked to the Evf (10.1601/nm.3165 virulence factor) protein [41]. The *evf* gene is present in the genome of 10.1601/nm.10935 SCC1 suggesting that the strain may have the ability to interact with insects.

## Conclusions

In this study, we presented the annotated genome sequence of the pectinolytic plant pathogen 10.1601/nm.10935 SCC1 consisting of a chromosome of 4,974,798 bp and a small cryptic plasmid of 5524 bp. Strain SCC1 was originally isolated from a diseased potato tuber and it has been used as a model strain to study interactions between soft rot pathogens and their host plants for decades. In accordance with the pathogenic lifestyle, the genome of strain SCC1 was found to harbor a large arsenal of plant cell wall-degrading enzymes similar to other sequenced 10.1601/nm.3241 genomes. In addition, an insect interaction gene, *evf*, is present in the genome of strain SCC1 suggesting the possibility of insects as vectors or alternative hosts for this strain. The genome sequence will drive further the use of 10.1601/nm.10935 SCC1 as a model plant pathogen.
